# Epicuticular lipids induce aggregation in Chagas disease vectors

**DOI:** 10.1186/1756-3305-2-8

**Published:** 2009-01-27

**Authors:** Alicia N Lorenzo Figueiras, Juan R Girotti, Sergio J Mijailovsky, M Patricia Juárez

**Affiliations:** 1 Laboratorio de Fisiología de Insectos, Dpto. Biodiversidad y Biología Experimental, Universidad de Buenos Aires, Ciudad Universitaria, Pabellón II, Ciudad Universitaria, 1428 Buenos Aires, Argentina; 2 Instituto de Investigaciones Bioquímicas de La Plata (CCT La Plata-CONICET-UNLP), Facultad de Ciencias Médicas, calles 60 y 120 1° piso, CP 1900, La Plata, Argentina

## Abstract

**Background:**

The triatomine bugs are vectors of the protozoan parasite *Trypanosoma cruzi*, the causative agent of Chagas disease. Aggregation behavior plays an important role in their survival by facilitating the location of refuges and cohesion of aggregates, helping to keep them safely assembled into shelters during daylight time, when they are vulnerable to predators. There are evidences that aggregation is mediated by thigmotaxis, by volatile cues from their faeces, and by hexane-extractable contact chemoreceptive signals from their cuticle surface. The epicuticular lipids of *Triatoma infestans *include a complex mixture of hydrocarbons, free and esterified fatty acids, alcohols, and sterols.

**Results:**

We analyzed the response of *T. infestans *fifth instar nymphs after exposure to different amounts either of total epicuticular lipid extracts or individual lipid fractions. Assays were performed in a circular arena, employing a binary choice test with filter papers acting as aggregation attractive sites; papers were either impregnated with a hexane-extract of the total lipids, or lipid fraction; or with the solvent. Insects were significantly aggregated around papers impregnated with the epicuticular lipid extracts. Among the lipid fractions separately tested, only the free fatty acid fraction promoted significant bug aggregation. We also investigated the response to different amounts of selected fatty acid components of this fraction; receptiveness varied with the fatty acid chain length. No response was elicited by hexadecanoic acid (C16:0), the major fatty acid component. Octadecanoic acid (C18:0) showed a significant assembling effect in the concentration range tested (0.1 to 2 insect equivalents). The very long chain hexacosanoic acid (C26:0) was significantly attractant at low doses (≤ 1 equivalent), although a repellent effect was observed at higher doses.

**Conclusion:**

The detection of contact aggregation pheromones has practical application in Chagas disease vector control. These data may be used to help design new tools against triatomine bugs.

## Background

The lipid layer on the insect cuticle comprises a complex mixture of hydrocarbons, free and esterified fatty acids and fatty alcohols, and smaller amounts of other oxygenated components [1,2]. Their role protecting insects from water loss and hence preventing lethal desiccation is widely recognized [3-5]. They are also the first barrier against chemical or biological contact insecticides [6,7]. Growing evidence has been gathered for more than 30 years on the role of hydrocarbons in chemical communication in many insect species [8]. However, relatively few reports are available on the participation of oxygenated cuticular lipid components on conspecific communication [9]. Among them, a methyl-branched ketone serves as the contact sex pheromone in the German cockroach [10], and an unsaturated ketone plays a similar role in the housefly [11]. Esterified fatty acids from honeybee larvae were reported to act as pheromones for honeybee workers and also as kairomones for parasitic mites [12,13]. The C16 and C18 acids were reported to participate in the chemical camouflage of a moth in honeybee colonies [14].

The epicuticular lipids of *Triatoma infestans *are mostly hydrocarbons, free and esterified fatty alcohols, triacylglycerols, free fatty acids, diacylglycerols, and small amounts of sterols, sterol esters, and monoacylglycerols [15]. The lipid composition was shown to vary quantitatively between juvenile and adult stages; and also with sex [16]. Cuticular hydrocarbon composition, structure and dynamics have been comprehensively studied [16-18]. Triacylglycerols, diacylglycerols, and free fatty acids hexane-extracted from the *T. infestans *cuticle show a similar pattern between adult and larvae in their fatty acid composition, prevailing not only the characteristic C14–C18 long chain fatty acids (palmitic, oleic, stearic, palmitoleic, linoleic, and myristic acids) but also variable amounts of fatty acids of very long chains. Among them, straight-chain saturated acids of 20–34 carbons were identified in the free acid fraction of adult insects and larvae, with C26 (hexacosanoic acid) as the major component [16].

In absence of other assembling chemical signals, i.e. faeces; behavioral assays showed that triatomine bugs aggregate on papers after contact with cuticular signals ('footprints') [19].

The aim of this study was to identify the major components of the contact aggregation pheromone in triatomines. We examined the effect of epicuticular lipid extracts, different lipid fractions, and free fatty acid components, on the aggregation behavior of *T. infestans *nymphs.

## Methods

### Insects

Our experiments were performed with *T. infestans *fifth instar nymphs reared at the INIBIOLP at 28°C, 50–60% relative humidity, under a 12 h dark- 12 h light photo cycle. All the insects were fed on chickens, 2–3 d after ecdysis, and used once, one week thereafter.

### Epicuticular extracts

Epicuticular lipids were extracted by immersing freeze-killed fifth instar *T. infestans *bugs in three successive portions of redistilled hexane (6 ml/g) for 5 minutes each [16]. The extracts were combined and concentrated under nitrogen. Separated lipid fractions were obtained by preparative thin layer chromatography (TLC) in silica gel plates (Aldrich, Milwaukee, WI, USA) after successive developments in hexane (100%), hexane: ethyl ether (95:5), and finally hexane: ethyl ether: acetic acid (80:20:1); all solvents were purchased from Carlo Erba, Milan, Italy. The major lipid fractions obtained were: hydrocarbons (HC), waxes (W), triacylglycerols (TG), diacylglycerols (DG), fatty alcohols (FA) and free fatty acids (FFA), as determined by comparison with standards similarly run, and as previously reported [15,18]. Lipid standards were from Sigma-Aldrich Co. (St. Louis, MO, USA), and Alltech Associates (Deerfield, IL, USA).

### Testing the activity of *T. infestans *epicuticular lipids, lipid fractions and selected fatty acid components

Hexane solutions of the epicuticular lipids, or each separate lipid fraction, were prepared in concentrations of 1 insect equivalent (E) in 40 μ1 of hexane. One insect equivalent of the total epicuticle lipids (or separate epicuticle lipid fraction) is the amount of total epicuticular lipids (or separate epicuticular lipid fraction) per insect. Because insects responded to their free fatty acid fraction (see Results), we also tested separately the major fatty acid components of this fraction: hexadecanoic acid (C16:0), octadecanoic acid (C18:0) and hexacosanoic acid (C26:0). The amount of each fatty acid tested was expressed in insect equivalents (one insect fatty acid equivalent is the amount of fatty acid present in the selected lipid fraction per insect) [15]. Hexane solutions of synthetic fatty acids (Sigma-Aldrich) were prepared and referred to insect equivalents according to the fatty acid composition and epicuticular lipid amounts of *T. infestans *nymphs previously reported [15].

To test the bioactivity of whole insect extracts, extract fractions, or separate free fatty acids, the appropriate hexane solutions were applied to the surface of Whatman #2 filter paper strips (2 × 3 cm) (Whatman Inc., New Jersey, USA). Control papers were similarly treated with same volume of hexane. The solvent was allowed to evaporate, and two treated papers (test and control) were carefully placed on a glass Petri dish arena (12 cm diameter) divided in two equal virtual sectors. Test and control papers were randomly placed in each sector to avoid location bias. Ten bugs were carefully released in the centre of each arena and videotaped for 1 h. After that period, the number of insects on each sector was counted. The number of replicates varied about 10 to 20 per dose tested. All glassware was cleaned with lab detergent, thoroughly rinsed, and oven dried before using. For each experimental series, the distribution of the insects in the arena was statistically analyzed by a G-test for goodness-of-fit. The null hypothesis was that the expected bug distribution followed a 1:1 ratio, considering an equal chance for bugs to stay either in the experimental (50%), or in the control paper (50%) [20]. The aggregation response to different fatty acid concentrations was compared by one factor ANOVA after arcsine transformation of the proportion values.

## Results

**Figure 1:**
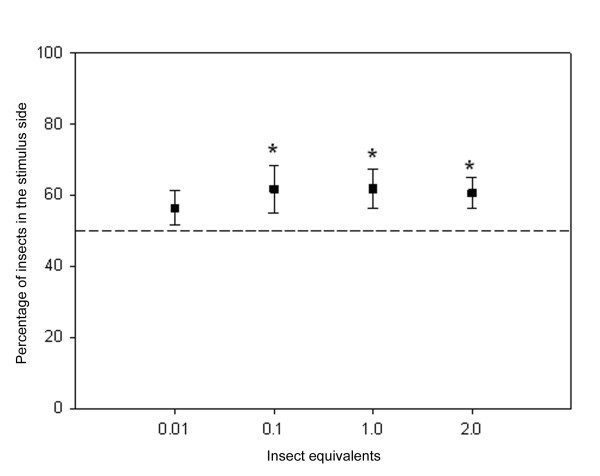
**Aggregation response of *T. infestans *fifth instar nymphs to the epicuticular lipid extracts of specimens of the same population (same age and instar)**. The dotted line indicates values expected for a random distribution. At 0.1, 1, and 2 equivalent doses, the response was significantly different from a random distribution. The numbers of replicates were N = 8 (0.1 insect equivalent), N = 18 (1 insect equivalent) and N = 17 (2 insect equivalents). Bars show standard error.

**Figure 2:**
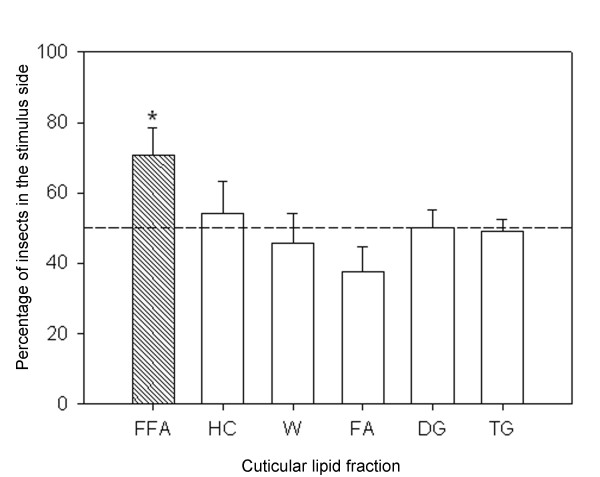
**Aggregation response (mean ± S.E.) of *T. infestans *fifth instar nymphs to 1 equivalent of the epicuticular lipid fractions of specimens of same age and instar**. DG: diacylglycerols, TG: triacylglycerols, W: waxes, FA: fatty alcohols, HC: hydrocarbons, FFA: free fatty acids. Asterisk indicates statistical significance. The number of replicates was N = 6, except for DG and TG (N = 7).

**Figure 3:**
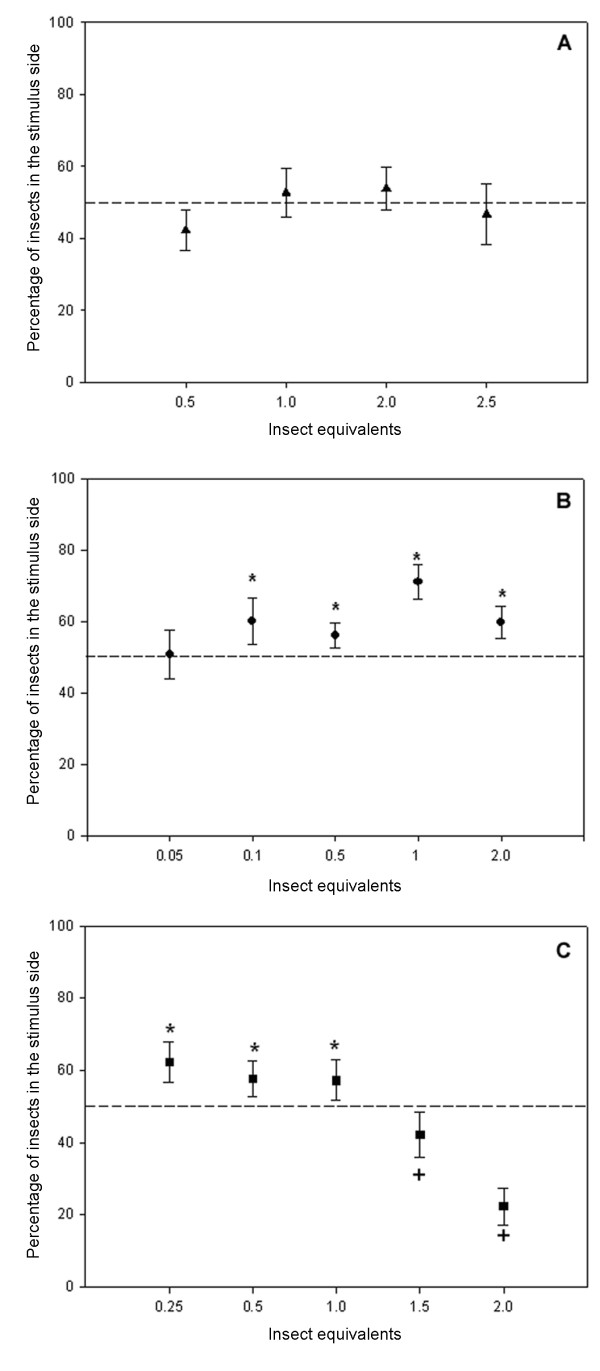
**Aggregation response (mean ± S.E.) of *T. infestans *larvae to different amounts of palmitic acid (C16:0) (A), stearic acid (C18:0) (B), and hexacosanoic acid (C26:0)**. Values for fatty acid equivalent were: 3 μg (C16:0), 2 μg (C18:0), 0.7 μg (C26:0). The number of replicates varied between N = 12–18 for each fatty acid.

Figure 1 shows the aggregation response of *T. infestans *fith instar nymphs to the epicuticular lipid extracts. Insects were significantly aggregated within the concentration range of 0.1 – 2 insect equivalents (G-test *P *≤ 0.001, *P *≤ 0.025 and *P *≤ 0.005 respectively). The epicuticular lipid extract was fractioned into the major lipid components by TLC, as described in Materials and Methods. TG, DG, FFA, FA, W and HC fractions were separately evaluated for aggregation response. Figure 2 shows the aggregation response of juvenile *T. infestans *bugs to 1 equivalent of each epicuticular lipid fraction obtained from specimens of similar age and instar. Only the FFA fraction promoted significant bug aggregation (*P *< 0.05). Among the major fatty acid components of this fraction, C16:0 showed a response not different from a random distribution within the dose range tested (0.5 – 2.5 insect equivalents) (Fig 3A). The assays performed with C18:0 showed a significant aggregation response between 0.1 – 2 insect equivalents (P < 0.025 for doses below 1 insect equivalent, and *P *< 0.001 for higher doses)(Fig. 3B). The very long chain fatty acid C26:0 elicited a significant attraction response between 0.25 – 1 insect equivalent (*P *< 0.05); however a significant repellency response was detected at higher doses (*P *< 0.05 for 1.5 insect equivalents and *P *< 0.001 for 2 insect equivalents) (Fig. 3C).

## Discussion

Aggregation pheromones consist of species-specific blends of chemicals causing conspecifics to increase their density in the vicinity of the pheromone source [21]. An heptane extract of onion maggot flies was reported as the aggregation pheromone released or left on the substrate by ovipositing females [22]. Among cuticular lipid components, hydrocarbons were reported to act as aggregation pheromones both in social and gregarious insects [23,24]. Cuticular extracts of the German cockroach *Blatella germanica *showed to act as an aggregation attractant cue, the cuticular hydrocarbons were detected as the major component responsible for this pheromone [25]. In the desert locust *Schistocerca gregaria*, the aggregation behavior of larvae is modulated by three sets of pheromonal compounds: aldehydes and acids emitted by the larvae themselves, and phenols associated with their feces [26]. In Heteroptera, the aggregation behavior of immature pentatomid bugs was investigated using cuticular extracts of six different pentatomid bug species, two aldehyde isomers elicited opposite responses, whereas the major cuticular hydrocarbon showed the highest attractiveness [27].

Research on model species from Blattodea, Coleoptera, Diptera, and Lepidoptera orders showed that many pheromones arise from modifications of fatty acid metabolism. For instance, the ketone contact sex pheromone of the female German cockroach, *Blattella germanica*, derives from elongation of a methyl-branched fatty acyl-CoA moiety followed by decarboxylation, hydroxylation, and oxidation [28,29]. The aggregation pheromone of *Tribolium castaneum *arises from a fatty acid precursor [30]. Also the major components of the sex pheromones in Diptera (e.g. *Drosophila *and *Muscidae*) are derived from elongation of fatty acyl-CoA moieties followed by loss of the carbonyl carbon and the formation of the corresponding hydrocarbon [31]. Lepidopteran sex pheromones are also derived from fatty acids, with diverse combinations of desaturation, chain-shortening, and modification of the carbonyl carbon [32,33]. Chemical communication activity has been reported for several esters of C16 and C18 fatty acids in the honeybee [12]. However, very few studies are available describing the aggregation role of insect fatty acids, among them; FFA components were reported as aggregation cues in the coleopteran *Trogodermes granarium *[34], and in social insects [35]. Also, endogenous free fatty acids were reported to be involved in conspecific attraction and food recognition in *Protaphorura armata *(Collembola) [36].

Our work is the first report providing evidence that cuticular lipid extracts of the kissing bug *T. infestans*, and particularly the C18:0 and C26:0 fatty acids components of the FFA fraction, promote insect aggregation. Aggregation behavior elicited by bug chemical signals has been proposed in several species of Triatominae, although no chemical identity was ever disclosed [37-40]. Furthermore, the first evidence that a hexane-extractable cuticular arresting signal promote aggregation through physical contact was reported in *T. infestans *juvenile stage [19].

In this study, cuticular lipid extracts elicited attraction response at all the doses tested (0.1–2.0 insect equivalents). Among major lipid components present in the epicuticle, the free fatty acid fraction was detected as the major responsible for this attraction (Fig. 2). Extracts of the behaviorally active cuticular lipids from *T. infestans *were early shown to contain a variety of fatty acids, either free (FFA) or esterified (TAG, DAG, MG, WE), among them the prevailing components were long chain (C16 and C18) and very long chain (C26) fatty acids [15]. Present data strongly indicates that certain FFA components are the cues responsible for the observed chemical attraction of *T. infestans *nymphs. Experiments with C16:0, C18:0 and C26:0 acids clearly showed that both C18:0 and C26:0 are involved in conspecific attraction, however C26:0 is attractive only at relatively low doses (≤ 1 insect equivalent); higher amounts produce a repellency effect, C16:0 *per se *does not participate in the aggregation response (Fig. 3). Whereas most FFA reported in insects usually range from C14 to C20 carbon number, very long chain fatty acids (VLCFA) have been rarely described in insect cuticular lipids, other than in the honeybee *Apis mellifera *[41] and in scale insect waxes [9]. In the frontal gland soldier secretions of the Formosan subterranean termite, C24:0 and C26:0 fatty acids were shown to exhibit a defensive role [42]. The repellency effect of the C26:0 fatty acid is far to be evident, although antimicrobial activity [43] and stimulation of the oxidation stress response in rats [44] have been also assigned to this VLCFA.

Further studies are needed in order to determine the optimal blend of fatty acids and cuticular components eliciting aggregation behavior in *T. infestans*, and whether these cues are attractant to the adult stage. Preliminary studies with bugs released into large size containers having conditioned cardboard boxes "painted" with cuticular extracts as the aggregation signal, showed an enhanced aggregation response (> 85%) in the longer term (12 h), compared to the 1-h experiments, reinforcing its potential utility for insect detection (Girotti and Juárez, unpublished results). Regarding Chagas disease transmission, both late nymph instars and adults exhibit the major vectorial capacity by potential *Trypanosoma cruzi *transmission through infective bite, hence they are the major targets in vector control strategies. Aggregation cues are important as potential components, in combination with host-related volatiles, in attractant trap devices to monitor Chagas disease vectors. Moreover, the implementation of new control tools is urgently needed for peridomestic environments where insecticide spraying is ineffective. This study provides evidence that *Triatoma *cuticle lipid components can be also promising candidates to be used in combination with insecticidal components in "trap and kill" devices.

## Competing interests

The authors have a patent pending on a blood-sucking insect trap, and a method to detect and control those insects.

## Authors' contributions

ANLF designed the bioassay studies, performed experiments, analyzed the data, and help to draft the manuscript. JRG and SJM performed experiments, carried out analysis of the data, and were involved in the drafting of the manuscript. MPJ conceived the study, participated in its design and coordination, and drafted the manuscript. All authors read and approved the final copy of this manuscript.
